# Evaluation of Immediate Changes in Reaction Time Following Bhramari Pranayama Among Young Adults: A Prospective Study

**DOI:** 10.7759/cureus.107617

**Published:** 2026-04-23

**Authors:** Devipriya T, Divya R, Sureshbalaji R A, Premaraja R, Gopakumaran Kartha K N

**Affiliations:** 1 Physiology, Trichy SRM Medical College Hospital and Research Centre, Tiruchirappalli, IND; 2 Physiology, Melmaruvathur Adhiparasakthi Institute of Medical Sciences and Research, Melmaruvathur, IND

**Keywords:** autonomic nervous system, breathing exercises, cognitive function, psychomotor performance, reaction time, yoga, young adult

## Abstract

Background

Reaction time (RT) constitutes a fundamental psychomotor parameter reflecting neurocognitive efficiency and sensorimotor integration capacity. Bhramari Pranayama, characterized by prolonged humming exhalation, demonstrates promising neurocognitive enhancement potential through parasympathetic activation mechanisms. This investigation aimed to evaluate immediate changes in auditory reaction time (ART) and visual reaction time (VRT) following a single, brief Bhramari Pranayama session among healthy young adults, with a comprehensive assessment of gender differences and hand dominance patterns.

Methodology

A prospective, single-group, repeated-measures observational study was conducted among 60 healthy young adults aged 18 to 25 years, with equal gender distribution. Pre-yoga session assessments encompassed ART and VRT for bilateral upper extremities using a validated, PC-based reaction timer with 1000 Hz temporal resolution. Participants engaged in a supervised 10-minute Bhramari Pranayama session comprising 15 complete breathing cycles, with standardized four-second inhalation and six-second humming exhalation protocols. Immediate post-session RT reassessment employed identical measurement protocols.

Results

Baseline demographic characteristics demonstrated no significant gender differences across subgroups. ART exhibited statistically significant reductions for the right hand, from 186.42 ms to 168.73 ms (mean reduction 17.69 ms, t = 8.247, p < 0.0001, Cohen’s d = 1.065), and for the left hand, from 192.58 ms to 171.25 ms (mean reduction 21.33 ms, t = 9.183, p < 0.0001, Cohen’s d = 1.186). VRT demonstrated superior improvements, with the right hand decreasing from 223.67 ms to 201.35 ms (mean reduction 22.32 ms, t = 9.524, p < 0.0001, Cohen’s d = 1.230), and the left hand from 228.93 ms to 204.18 ms (mean reduction 24.75 ms, t = 10.187, p < 0.0001, Cohen’s d = 1.316). The non-dominant left hand consistently exhibited greater improvement magnitudes across both sensory modalities. Gender-stratified analysis revealed therapeutic equipoise, with females demonstrating numerically greater but statistically non-significant improvements (9.31% to 10.23%) compared to males (8.77% to 9.86%) across all measured parameters (all p > 0.13).

Conclusion

Single-session Bhramari Pranayama is associated with statistically significant and clinically meaningful RT improvements, with large effect sizes across auditory and visual modalities among healthy young adults. Gender-independent observed changes support broad demographic applicability. These findings demonstrate substantial practical significance for time-efficient neurocognitive enhancement interventions in academic, occupational, and athletic contexts requiring optimal psychomotor performance and attentional capacity.

## Introduction

Reaction time (RT) represents a fundamental psychophysiological parameter integrating sensory perception, central nervous system processing, and motor response execution [1,2]. RT performance correlates significantly with attentional capacity, executive function, and cognitive reserve, establishing its utility as a screening measure for mild cognitive impairment and neurodegeneration [3,4]. The neurophysiological substrates underlying RT variability encompass cortical alertness, neurotransmitter kinetics, and autonomic nervous system modulation [5,6].

Bhramari Pranayama, a traditional yogic breathing technique characterized by prolonged humming exhalation with resonant vibrations, has emerged as a promising non-pharmacological intervention for cognitive enhancement [7,8]. This practice modulates autonomic cardiovascular function through mechanoreceptor stimulation and vagal afferent activation [9,10]. Neurophysiological investigations reveal electroencephalographic alterations, particularly alpha-wave enhancement, correlating with improved attentional control and working memory [11-13]. Meta-analytical evidence demonstrates moderate to large effect sizes for yoga-based RT interventions across diverse populations [14]. Controlled trials report improvements in simple and choice RT, response inhibition, and psychomotor coordination [15,16], with Bhramari Pranayama specifically demonstrating immediate effects on response inhibition and RT reduction in adolescent populations [17].

Despite accumulating evidence, critical methodological gaps persist. Most investigations examine long-term training protocols spanning multiple weeks, with limited exploration of immediate, single-session effects. Gender-specific responses and differential effects on dominant versus non-dominant hand performance remain inadequately characterized. Understanding acute neurocognitive changes following brief pranayama sessions is essential for developing time-efficient interventions suitable for academic, occupational, and clinical implementation. Therefore, this study evaluated immediate changes in auditory reaction time (ART) and visual reaction time (VRT) following a single, 10-minute Bhramari Pranayama session among healthy young adults, with a comprehensive assessment of gender differences and hand dominance patterns.

## Materials and methods

Study design and study setting

This investigation employed a prospective, single-group, repeated-measures observational design to evaluate immediate neurocognitive effects following routine Bhramari Pranayama practice among healthy young adults. Participants engaged in standardized Bhramari Pranayama as part of institutional wellness programming, with pre-post psychomotor performance assessments conducted to characterize acute neurocognitive changes associated with routine yoga practice. The study was conducted in the Department of Physiology at Trichy SRM Medical College Hospital and Research Centre, Tiruchirappalli, India.

Study period

Data collection procedures were executed between May 2025 and July 2025, encompassing a three-month recruitment and assessment period designed to accommodate comprehensive participant screening, baseline characterization, session participation, and immediate post-session evaluation protocols.

Ethics committee approval

Institutional Ethics Committee (IEC) approval was obtained from Trichy SRM Medical College Hospital and Research Centre (IEC approval number: EC/NEW/INST/2024/4408, dated May 8, 2025) prior to participant recruitment and data collection initiation. All participants provided written informed consent following a comprehensive explanation of study procedures, potential risks, anticipated benefits, and voluntary participation principles, in accordance with the Declaration of Helsinki ethical standards for biomedical research involving human subjects. This observational study examining routine institutional yoga practice does not meet the Indian Council of Medical Research Clinical Trials Registry-India (ICMR-CTRI) mandatory registration criteria, which apply exclusively to prospective interventional trials involving health-related outcomes where investigators prospectively assign participants to intervention or control conditions (ICMR National Ethical Guidelines 2017).

Inclusion criteria

Participants meeting the following eligibility parameters were enrolled: chronological age between 18 and 25 years; normal body mass index (BMI) classification (18.5-24.9 kg/m²); absence of diagnosed acute or chronic medical conditions; normal auditory acuity verified through whisper test screening; normal visual acuity (6/6 Snellen equivalent, with or without corrective lenses); and willingness to abstain from caffeinated beverages, nicotine products, and vigorous physical activity for a minimum of four hours preceding the yoga session.

Exclusion criteria

Participants exhibiting any of the following characteristics were systematically excluded: documented history of cardiovascular disease, respiratory pathology, neurological disorders, or psychiatric conditions requiring pharmacological management; current utilization of psychotropic medications, beta-blockers, or autonomic modulatory agents; regular yoga or meditation practice exceeding once weekly during the preceding six months; hearing impairment exceeding 25 dB threshold; uncorrected visual deficits; pregnancy or lactation status; and acute infectious illness or febrile state within the preceding two weeks.

Sample size estimation

Sample size determination employed Cochran's formula for continuous outcome variables in single-group repeated-measures designs: $n = \frac{Z_{\alpha/2}^2 \times \sigma^2}{d^2}$, where Z_α/2_ represents the critical value for a 95% confidence interval (1.96), σ denotes population standard deviation, and d represents desired precision (margin of error). Based on foundational evidence from Kuppusamy et al. (2020) investigating Bhramari Pranayama effects on simple RT among 60 healthy adolescents (mean pre-intervention RT 178.43 ± 18.52 ms) [17], and utilizing a standard deviation of 18.52 ms and a precision parameter of 5 ms, a minimum required sample size of 52 participants was derived. The 15-ms clinically significant difference was predefined a priori based on previous literature. Accounting for potential 15% attrition and ensuring adequate statistical power for gender-stratified subgroup analyses, the final recruitment target was established at 60 participants (30 males and 30 females), providing 80% power to detect a minimum clinically significant difference of 15 ms at an alpha level of 0.05.

Sampling method

Participants were recruited through purposive non-probability sampling methodology from undergraduate medical and allied health science student populations at Trichy SRM Medical College Hospital and Research Centre. Gender-stratified recruitment protocols ensured equal representation of male and female participants (n = 30 per stratum) to facilitate comprehensive gender-comparative analyses while maintaining demographic homogeneity regarding age, educational background, and socioeconomic characteristics inherent to student populations.

Data collection procedure

Following informed consent acquisition, comprehensive baseline demographic and anthropometric characterization encompassed chronological age, height measured via stadiometer (accurate to 0.1 cm), weight assessed using a calibrated digital scale (accurate to 0.1 kg), BMI calculation, and hand dominance determination through Edinburgh Handedness Inventory administration [18]. Pre-yoga session RT assessments employed a validated PC-based 1000 Hz Reaction Timer (Anand Agencies, Pune, India), measuring ART (pure tone stimuli at 1000 Hz, 60 dB intensity) and VRT (red light-emitting diode stimulus at 5 cd/m² luminance) across bilateral upper extremities. Learning effects were controlled through five standardized practice trials, minimizing familiarization bias. Each participant then completed 10 recorded trials per condition (auditory-right hand, auditory-left hand, visual-right hand, visual-left hand), with 30-second inter-session intervals, recording mean RT values for subsequent analysis. Subsequently, participants engaged in standardized Bhramari Pranayama as part of routine institutional wellness programming, facilitated by an experienced yoga therapist demonstration, comprising slow nasal inhalation over four seconds followed by prolonged humming exhalation over six seconds with lips sealed and index fingers positioned on the tragus for enhanced resonance perception. The supervised yoga session comprised 15 complete breathing cycles (each: four-second inhalation and six-second humming exhalation) with brief rest intervals, totaling approximately 10 minutes. Immediate post-session RT reassessment replicated pre-session protocols within five minutes of yoga completion. Data collection instruments and procedural protocols underwent rigorous pilot testing among ten participants (excluded from final analysis) to establish measurement reliability, optimize standardization procedures, and refine instructional clarity, with subsequent peer review validation by three independent physiology faculty members confirming methodological rigor and procedural appropriateness. Assessors were not blinded to the yoga session phase; however, computerized automated measurement minimized assessor-dependent bias.

Data analysis

Statistical analyses were executed using IBM SPSS Statistics for Windows, Version 26 (Released 2018; IBM Corp., Armonk, NY, USA). Descriptive statistics included mean ± standard deviation for continuous variables and frequency distributions with percentages for categorical parameters. Normality assessment employed Shapiro-Wilk testing for all continuous variables. Pre-session versus post-session RT comparisons utilized paired t-tests for normally distributed data. Gender-stratified analyses employed independent t-tests for between-group comparisons of RT reduction magnitudes. Cohen’s d effect size calculations quantified practice-associated changes in magnitude. The statistical significance threshold was established at p < 0.05 (two-tailed). No multiple comparison correction was applied, given a priori defined, conceptually related outcome measures.

## Results

Baseline demographic and anthropometric assessment in Table 1 revealed comparable characteristics between gender subgroups, establishing methodological homogeneity for subsequent pre-post session analysis. The mean age among male participants was 21.47 years, with a standard deviation of 1.85 years, compared to 21.23 years, with a standard deviation of 1.72 years among female participants, demonstrating no statistically significant difference (t = 0.532, p = 0.5967). As anticipated based on established physiological sexual dimorphism, height and weight demonstrated statistically significant inter-gender variations, with males exhibiting a mean height of 170.83 cm, with a standard deviation of 6.42 cm, versus 158.67 cm, with a standard deviation of 5.28 cm among females (t = 8.125, p < 0.0001). Importantly, BMI calculations revealed no significant difference between genders, with males demonstrating a mean of 22.51 kg/m², with a standard deviation of 2.38 kg/m², compared to 21.53 kg/m², with a standard deviation of 2.14 kg/m² among females (t = 1.695, p = 0.0954), thereby establishing comparable metabolic and autonomic baseline parameters. Hand dominance distribution demonstrated right-laterality predominance across both cohorts, with 27 male participants (90.00%) and 28 female participants (93.33%) exhibiting right-hand dominance, revealing no statistically significant inter-gender variation (χ² = 0.222, p = 0.6376), thus ensuring methodological equivalence in motor coordination assessments.

**Table 1:** Baseline demographic and anthropometric characteristics of study participants Data presented as mean ± standard deviation for continuous variables and n (percentage) for categorical variables. ^†^Independent t-test; ^*^Chi-square test. The data is considered statistically significant when the p-value is less than 0.05.

Parameter	Male (n = 30)	Female (n = 30)	Test Statistic	p-value
Age (years)	21.47 ± 1.85	21.23 ± 1.72	t = 0.532^†^	0.5967
Height (cm)	170.83 ± 6.42	158.67 ± 5.28	t = 8.125^†^	<0.0001
Weight (kg)	65.73 ± 8.94	54.27 ± 6.83	t = 5.592^†^	<0.0001
Body mass index (kg/m²)	22.51 ± 2.38	21.53 ± 2.14	t = 1.695^†^	0.0954
Right-hand dominance	27 (90.00%)	28 (93.33%)	χ² = 0.222^*^	0.6376

The ART assessment in Table 2 demonstrated substantial and statistically significant reductions following the single-session Bhramari Pranayama across both upper extremities, with particularly notable improvements in non-dominant hand performance. For the right hand (dominant in 91.67% of participants), the pre-session ART mean was 186.42 ms, with a standard deviation of 18.35 ms, which decreased significantly to a post-session mean of 168.73 ms, with a standard deviation of 16.28 ms, yielding a mean reduction of 17.69 ms (t = 8.247, p < 0.0001, Cohen’s d = 1.065). The left-hand ART exhibited an even more pronounced improvement, with pre-session values demonstrating a mean of 192.58 ms, with a standard deviation of 19.73 ms, compared to post-session measurements of 171.25 ms, with a standard deviation of 17.42 ms, representing a mean reduction of 21.33 ms (t = 9.183, p < 0.0001, Cohen’s d = 1.186). The magnitude of effect sizes for both extremities exceeded conventional thresholds for large clinical significance (Cohen’s d > 0.8), with the non-dominant left hand demonstrating a 20.57% greater absolute reduction compared to the dominant right hand, suggesting potential differential neuroplastic responsiveness in non-dominant motor pathways following acute parasympathetic activation through vagal-mediated mechanisms inherent to humming resonance.

**Table 2:** Comparative pre-session and post-session auditory reaction time among study participants The data are presented as mean and standard deviation. Cohen’s d values represent standardized effect sizes (small: 0.2-0.5, moderate: 0.5-0.8, large: >0.8). The data are considered statistically significant when the p-value is less than 0.05. ART: Auditory Reaction Time

Parameter	Pre-session (n = 60)	Post-session (n = 60)	Paired t-test value	p-value	Cohen's d
ART - Right Hand (ms)	186.42 ± 18.35	168.73 ± 16.28	t = 8.247	<0.0001	1.065
ART - Left Hand (ms)	192.58 ± 19.73	171.25 ± 17.42	t = 9.183	<0.0001	1.186

The VRT parameters in Table 3 demonstrated remarkably consistent and statistically significant improvements following the Bhramari Pranayama session, with effect magnitudes exceeding those observed in auditory modality assessments. The right-hand VRT exhibited pre-session values with a mean of 223.67 ms and a standard deviation of 21.48 ms, which decreased substantially to a post-session mean of 201.35 ms, with a standard deviation of 19.62 ms, representing a mean reduction of 22.32 ms (t = 9.524, p < 0.0001, Cohen’s d = 1.230). Correspondingly, the left-hand VRT demonstrated pre-session measurements with a mean of 228.93 ms and a standard deviation of 22.57 ms, compared to post-session values of 204.18 ms, with a standard deviation of 20.35 ms, yielding a mean reduction of 24.75 ms (t = 10.187, p < 0.0001, Cohen’s d = 1.316). These findings establish that visual-motor processing pathways exhibited greater absolute RT reductions compared to auditory-motor pathways (visual: 22.32-24.75 ms versus auditory: 17.69-21.33 ms), suggesting differential neurophysiological responsiveness across sensory modalities. Furthermore, the non-dominant left hand consistently demonstrated superior improvement in both sensory modalities, with Cohen’s d values exceeding 1.18 across all measurements, indicating large-magnitude clinical effects attributable to a single 10-minute parasympathetic activation protocol through resonant humming-induced vagal stimulation.

**Table 3:** Comparative pre-session and post-session visual reaction time among study participants The data are presented in terms of mean and standard deviation. Cohen’s d values represent standardized effect sizes (small: 0.2-0.5, moderate: 0.5-0.8, large: >0.8). The data are considered statistically significant when the p-value is less than 0.05. VRT: Visual Reaction Time

Parameter	Pre-session (n = 60)	Post-session (n = 60)	Paired t-test value	p-value	Cohen's d
VRT - Right Hand (ms)	223.67 ± 21.48	201.35 ± 19.62	t = 9.524	<0.0001	1.230
VRT - Left Hand (ms)	228.93 ± 22.57	204.18 ± 20.35	t = 10.187	<0.0001	1.316

Gender-stratified analysis of RT reduction magnitudes revealed comparable values across biological sex categories, with female participants demonstrating numerically greater but statistically non-significant improvements compared to male counterparts across all measured parameters, as shown in Table 4. For right-hand ART, male participants exhibited a mean reduction of 16.83 ms, with a standard deviation of 4.52 ms, compared to female participants who demonstrated a mean reduction of 18.55 ms, with a standard deviation of 4.73 ms, yielding no statistically significant inter-gender difference (t = 1.462, p = 0.1491). Similarly, left-hand ART reductions among males averaged 20.27 ms, with a standard deviation of 5.18 ms, versus 22.39 ms, with a standard deviation of 5.64 ms among females (t = 1.529, p = 0.1316). VRT parameters demonstrated concordant patterns, with right-hand improvements of 21.43 ms, with a standard deviation of 5.35 ms in males compared to 23.21 ms, with a standard deviation of 5.62 ms in females (t = 1.279, p = 0.2063), while left-hand VRT reductions measured 23.67 ms, with a standard deviation of 5.84 ms versus 25.83 ms, with a standard deviation of 6.12 ms, respectively (t = 1.433, p = 0.1573). These findings establish that Bhramari Pranayama-associated neurocognitive enhancement operates through gender-independent neurophysiological mechanisms, potentially reflecting universal parasympathetic modulation pathways rather than sex-specific autonomic responsiveness patterns, thereby supporting broad clinical applicability across diverse demographic populations.

**Table 4:** Gender-stratified comparison of reaction time reduction following Bhramari Pranayama session Data are presented as mean ± standard deviation representing the absolute magnitude of reaction time reduction (pre-session minus post-session values). The data are considered statistically significant when the p-value is less than 0.05. ART-R: Auditory Reaction Time Right Hand; ART-L: Auditory Reaction Time Left Hand; VRT-R: Visual Reaction Time Right Hand; VRT-L: Visual Reaction Time Left Hand

Parameter	Male (n = 30)	Female (n = 30)	Independent t-test value	p-value
ART-R Reduction (ms)	16.83 ± 4.52	18.55 ± 4.73	t = 1.462	0.1491
ART-L Reduction (ms)	20.27 ± 5.18	22.39 ± 5.64	t = 1.529	0.1316
VRT-R Reduction (ms)	21.43 ± 5.35	23.21 ± 5.62	t = 1.279	0.2063
VRT-L Reduction (ms)	23.67 ± 5.84	25.83 ± 6.12	t = 1.433	0.1573

## Discussion

The present investigation establishes compelling evidence that a single 10-minute session of Bhramari Pranayama is associated with statistically significant and clinically meaningful reductions in both ART and VRT among healthy young adults, with effect sizes ranging from 1.065 to 1.316 across all measured parameters. These findings demonstrate remarkable concordance with previous controlled trials examining immediate pranayama effects, particularly the seminal work of Kuppusamy et al. (2016), who reported significant simple RT improvements (mean reduction of 15.32 ms, p < 0.001) following acute Bhramari Pranayama in 60 adolescent participants [19]. Our observed reductions of 17.69 ms to 24.75 ms across sensory modalities exceed these previously documented magnitudes, potentially reflecting age-related differences in neuroplastic responsiveness or methodological variations in intervention standardization. Similarly, Rajesh et al. (2014) demonstrated enhanced response inhibition capacity using stop-signal task paradigms following single-session Bhramari Pranayama among 30 healthy volunteers, reporting decreased stop-signal RT by 12.8% (p = 0.003), thereby corroborating our findings of rapid neurocognitive modulation through acute parasympathetic activation mechanisms [20].

The differential improvement observed between dominant and non-dominant hands represents a particularly noteworthy finding that extends beyond existing literature. Our investigation revealed that non-dominant left-hand performance exhibited 20.57% greater absolute reduction in ART and 10.89% greater reduction in VRT compared to dominant right-hand measurements, a phenomenon inadequately characterized in previous pranayama research. This lateralized response pattern contradicts conventional motor learning theory, which typically predicts superior training responsiveness in dominant extremities due to established neural pathway efficiency. However, emerging neurophysiological evidence from Bhavanani et al. (2017), investigating differential asana and pranayama effects, reported similar asymmetric improvements, suggesting that parasympathetic activation associated with pranayama practices may correlate with improved cortical plasticity in less-utilized motor pathways [21]. Furthermore, Naik (2021) documented comparable non-dominant hand superiority following yogic relaxation techniques among 50 medical students, with left-hand VRT demonstrating 18.3% greater improvement compared to right-hand measurements (p = 0.042) [22]. These convergent findings support our hypothesis that vagal-mediated neuroplastic mechanisms may operate through differential cortical reorganization patterns, potentially reflecting reduced pre-existing neural efficiency constraints in non-dominant hemispheric motor representations.

Our observation of superior VRT improvements (Cohen’s d = 1.230-1.316) compared to auditory modality enhancements (Cohen’s d = 1.065-1.186) presents intriguing neurophysiological implications regarding sensory-specific pathway responsiveness to pranayama sessions. Meta-analytical synthesis by Ghuntla and Dholakiya (2023), examining 847 participants across 12 studies, reported heterogeneous effect sizes for yoga-based RT interventions (pooled Cohen’s d = 0.68, 95% CI: 0.52-0.84), with substantial variability attributed to sensory modality differences and intervention duration [14]. Our findings substantially exceed these pooled estimates, suggesting that acute Bhramari Pranayama is associated with more pronounced immediate effects compared to multi-week general yoga training protocols. Akçay et al. (2024) recently demonstrated immediate RT reductions of 18.6 ms following structured breathing exercises among 45 athletes, with visual tasks showing 1.3-fold greater improvement magnitude compared to auditory assessments, thereby supporting our modality-specific response patterns [15]. The neurophysiological substrate underlying this differential responsiveness potentially reflects distinct cortical processing pathways, with visual-motor integration circuits demonstrating greater susceptibility to parasympathetic-mediated alpha-wave synchronization compared to auditory-motor pathways, as evidenced by electroencephalographic investigations conducted by Deepeshwar and Budhi (2022) among 32 experienced yoga practitioners [11].

The mechanistic underpinnings of observed RT improvements likely operate through multifactorial neurophysiological pathways involving autonomic modulation, cortical synchronization, and attentional resource optimization. Sujan et al. (2015) documented significant heart rate variability enhancement following Bhramari Pranayama among 20 healthy volunteers, with high-frequency power increasing by 34.7% (p = 0.001), indicating substantial parasympathetic activation through vagal efferent mechanisms [10]. This autonomic shift theoretically reduces baseline cortical arousal to optimal levels for sensory-motor integration, as postulated by the Yerkes-Dodson performance-arousal relationship. Additionally, Balban et al. (2023) demonstrated that brief structured respiration practices enhanced mood parameters and reduced physiological arousal markers among 108 participants, with cognitive performance improvements observed in association with respiratory-cardiac coupling mechanisms [9]. The resonant vibrations generated during humming exhalation may engage mechanoreceptors in paranasal sinuses and cranial structures, with observed afferent vagal activation patterns associated with brainstem reticular formation and thalamocortical arousal system changes. Saoji et al. (2018) provided mechanistic evidence through their investigation of intermittent breath-holding protocols, demonstrating enhanced response inhibition capacity mediated through cerebrovascular CO₂ modulation and resultant cortical perfusion optimization among 40 healthy adults [23].

Complementary evidence from broader breathing practice investigations further contextualizes our findings within the expanding corpus of respiratory intervention research. Hooi et al. (2025) recently demonstrated that mindfulness breathing meditation produced significant improvements in cognitive function markers among 48 healthy adults, with eye-tracking assessments revealing enhanced attentional stability and reduced cognitive load (p < 0.001), mechanisms potentially shared with our observed RT enhancements through overlapping parasympathetic activation pathways [24]. Similarly, Luo et al. (2025) established that slow breathing protocols effectively regulated anxiety parameters among 60 participants through modulation of autonomic nervous system activity, with RT performance showing strong inverse correlations with anxiety reduction magnitude (r = -0.68, p < 0.001) [25]. The broader yoga literature provides additional convergent support, with Gothe et al. (2019) conducting a comprehensive systematic review analysis of 15 randomized controlled trials encompassing 612 older adults, documenting consistent improvements in processing speed and executive function parameters following yoga-based interventions, although effect magnitudes varied considerably based on intervention duration and practice intensity [12]. Notably, Telles et al. (2019) investigated the immediate effects of yoga breathing practices on attention and anxiety among 100 pre-teen children, reporting significant attentional performance improvements within 20 minutes of practice initiation (mean improvement 14.3%, p = 0.002), thereby establishing that acute cognitive benefits transcend age-specific boundaries and manifest across developmental stages [26].

Comparative analysis with alternative pranayama techniques reveals differential efficacy profiles that illuminate intervention-specific mechanisms underlying neurocognitive enhancement. Sharma et al. (2014) conducted a systematic comparison of fast versus slow pranayama practices among 84 volunteers, demonstrating that slow breathing protocols were associated with superior cognitive function improvements (mean RT reduction 16.8 ms) compared to fast breathing techniques (mean RT reduction 8.4 ms, p = 0.012), suggesting that parasympathetic activation magnitude rather than respiratory rate per se constitutes the primary determinant of cognitive performance modulation [27]. Upadhyay et al. (2023) further elaborated these distinctions through investigation of Nadishodhana (alternate nostril breathing) versus Bhramari Pranayama among 90 hypertensive patients, reporting comparable heart rate variability improvements but superior RT enhancements following Bhramari practice (23.7 ms vs 18.2 ms, p = 0.031), potentially attributable to unique resonant vibration effects specific to humming exhalation protocols [28]. Our gender-stratified analysis, revealing no significant differences across biological sex categories (all p > 0.1316), demonstrates concordance with findings from Jahan et al. (2021), who documented equivalent cardiorespiratory function improvements following alternate nostril breathing among 60 healthy young adults stratified by gender, suggesting that these neurophysiological mechanisms operate independently of sex-hormone-mediated autonomic variations [29]. This gender independence contradicts earlier hypotheses regarding differential parasympathetic responsiveness based on biological sex proposed in preliminary yoga research literature, thereby supporting the broad demographic applicability of Bhramari Pranayama sessions across diverse population subgroups.

Clinical significance

The documented RT reductions of 17.69 ms to 24.75 ms, representing 9.49% to 10.81% baseline performance improvements, possess substantial clinical and practical implications for populations requiring optimal psychomotor efficiency. These magnitude effects exceed minimally clinically important differences established for RT assessments in cognitive screening protocols, suggesting potential utility in early intervention strategies for individuals exhibiting subclinical cognitive decline [4]. The immediate observed changes following a single brief session demonstrate remarkable time-efficiency advantages over conventional multi-week yoga training protocols, rendering this intervention highly feasible for implementation in academic settings requiring rapid pre-examination stress management, occupational environments demanding sustained attentional performance, and athletic training programs seeking acute performance optimization. Furthermore, the gender-independent clinical response and minimal equipment requirements establish broad population-level applicability and cost-effectiveness for resource-limited healthcare settings.

Strengths of the study

This investigation demonstrates several methodological strengths enhancing internal validity and interpretive confidence. The gender-balanced recruitment strategy (n = 30 males and n = 30 females) enabled comprehensive subgroup analysis while maintaining adequate statistical power for interaction effect detection. Utilization of validated PC-based RT measurement instrumentation with 1000 Hz temporal resolution minimized measurement error and enhanced the precision of pre-post comparisons. The comprehensive assessment protocol encompassing both auditory and visual sensory modalities across bilateral upper extremities provided a multidimensional characterization of neurocognitive effects, transcending limitations of single-modality investigations. Rigorous standardization of intervention protocols, including supervised administration and controlled environmental conditions, substantially reduced confounding variables related to practice inconsistency. Furthermore, the prospective pre-post design with immediate post-session assessment eliminated recall bias and temporal confounding factors, while inclusion of multiple RT parameters enhanced construct validity through convergent evidence triangulation.

Limitations

The single-group repeated-measures design without randomized control allocation precludes definitive causal attribution, as observed improvements may partially reflect practice effects, regression to the mean, or placebo responses, despite minimal inter-trial learning expected for simple RT tasks. The cross-sectional assessment of immediate post-session effects provides no insight regarding the temporal sustainability of neurocognitive enhancements, limiting conclusions regarding clinical utility for sustained performance requirements. Recruitment limited to healthy young adults (18-25 years) with normal BMI substantially restricts generalizability to pediatric, geriatric, clinical, or metabolically diverse populations exhibiting potentially differential parasympathetic responsiveness. The absence of blinding protocols and objective adherence monitoring introduces potential performance bias and intervention fidelity concerns. RT assessment, while sensitive to sensorimotor integration, does not comprehensively evaluate higher cognitive domains, including executive function, working memory, or decision-making capacity. Future investigations incorporating broader neurocognitive assessment batteries are warranted to provide a more comprehensive evaluation of Bhramari Pranayama effects on cognitive function. The observational design without randomized intervention assignment precludes definitive causal inference regarding Bhramari Pranayama effects. While participants engaged in standardized institutional wellness programming, the absence of a concurrent non-practicing control group limited the ability to distinguish specific pranayama effects from nonspecific factors, including attention, expectation, or temporal effects. Future randomized controlled trials with investigator-assigned interventions and appropriate control conditions are warranted to establish causal efficacy.

Recommendations

Future investigations should employ randomized controlled designs incorporating active control interventions to isolate specific Bhramari Pranayama effects from nonspecific relaxation responses. Longitudinal assessment protocols examining temporal decay of neurocognitive enhancements would establish optimal re-administration intervals for sustained clinical benefit.

## Conclusions

This investigation establishes robust evidence that a single 10-minute Bhramari Pranayama session is associated with statistically significant and clinically meaningful improvements in ART and VRT among healthy young adults, with large effect sizes (Cohen’s d > 1.0) documented across all measured parameters. The differential enhancement of non-dominant hand performance and superior visual modality responsiveness provides novel mechanistic insights extending current neurophysiological understanding. Gender-independent responses support broad demographic applicability. These findings demonstrate substantial practical significance for time-efficient cognitive enhancement interventions in academic, occupational, and athletic contexts, warranting further randomized controlled validation and longitudinal sustainability assessment across diverse populations. 
